# A Regional Approach to Malaria Molecular Surveillance in Southern Africa: Lessons from the GenE8 Initiative

**DOI:** 10.4269/ajtmh.25-0130

**Published:** 2026-07-14

**Authors:** Jaishree Raman, Mukosha Chisenga, Chadwick Sikaala, Andrés Aranda-Díaz, Allison Tatarsky, Roly Gosling, John Chimumbwa, Jennifer L. Smith

**Affiliations:** ^1^ Centre for Emerging Zoonotic and Parasitic Diseases, National Institute for Communicable Diseases, Johannesburg, South Africa;; ^2^ Wits Research Institute for Malaria, University of Witwatersrand, Johannesburg, South Africa;; ^3^ Institute for Sustainable Malaria Control, University of Pretoria, Pretoria, South Africa;; ^4^ SADC Malaria Elimination 8 Secretariat, Windhoek, Namibia;; ^5^ EPPIcenter Research Program, Department of Medicine, University of California San Francisco, San Francisco, California, USA;; ^6^ Malaria Elimination Initiative, University of California San Francisco, San Francisco, California, USA;; ^7^ ISGlobal, Barcelona, Spain;; ^8^ London School for Hygiene and Tropical Medicine, London, United Kingdom;; ^9^ Department of Epidemiology and Biostatistics, University of California San Francisco, San Francisco, California, USA

## Abstract

The growing threat of artemisinin partial resistance prompted the WHO to recommend routine monitoring of drug efficacy and resistance. Advances in sequencing technologies and expanded laboratory capacity across Africa have made malaria molecular surveillance (MMS) a viable option for many countries. Despite improved access to next-generation sequencing platforms, the uptake of MMS for programmatic decision-making and the availability of antimalarial resistance marker data in southern Africa have lagged behind those in other regions. The Genomics of Malaria in the Elimination Eight (GenE8) initiative implemented a regional MMS model comprising 1) regional consensus-building and governance, 2) capacity strengthening, and 3) evidence generation. Through this initiative, standardized parasite genomic data across five southern African countries (Angola, Eswatini, Namibia, South Africa, and Zambia) were generated using a targeted amplicon deep-sequencing workflow. Regional laboratory and analytical skills were strengthened, and national malaria programs (NMPs) were capacitated to use genomic data for decision-making through a regional MMS fellowship and training workshops. Although the initiative achieved important successes, persistent challenges limited its overall impact despite the implementation of integrated mitigation strategies. Challenges included constraints in regional bioinformatics capacity, delays in sample shipment and cross-border data sharing, and a lack of sustained funding. In the present case study, the GenE8 model, operational experiences, and cross-cutting lessons learned are described. The GenE8 experience reveals that regional MMS is feasible in southern Africa and that near-real-time resistance data can inform policy and intervention decisions, but that it requires sustained investment in governance mechanisms, bioinformatics expertise, and institutionalized NMP capacity to be sustainable.

## INTRODUCTION

Recent advances in genotyping technologies and increased accessibility after the coronavirus disease 2019 (COVID-19) pandemic have made integrating next-generation sequencing (NGS) into routine malaria surveillance more feasible and cost-effective for national malaria programs (NMPs) and public health research institutes (PHRIs).^1^ Through the efforts of the WHO, the Africa Centers for Disease Control (CDC),^2^ and partners, NGS capacity had expanded to 53 of the 55 African Union states as of 2024.^3^ Unfortunately, in southern Africa, this expansion of in-country NGS capacity has not translated into increased availability or routine use of malaria parasite genomic data by NMPs.

Malaria molecular surveillance (MMS) is the public health application of NGS-generated parasite genomic data for surveillance and decision-making. Accurate molecular data on parasite population structure and resistant allele prevalence can fill critical gaps in routine malaria surveillance data and inform intervention targeting, product choice, and efficacy testing.^4^ Malaria molecular surveillance is increasingly used as an early warning system for detecting antimalarial, insecticide, and diagnostic resistance and can support other use cases such as monitoring intervention efficacy, mapping parasite connectivity, and distinguishing locally acquired from imported infections, which is critical information for NMPs in lower-transmission settings.^5^

Despite the growing applications, the implementation of MMS within routine malaria programs remains limited. Much of the technical expertise and analytical capacity required for genomic surveillance in Africa resides in academic institutions,^1^ whereas NMPs and partner PHRIs have limited internal capacity to generate, analyze, interpret, and use genomic data for programmatic decision-making. Infrastructure for NGS also remains expensive to establish and maintain, requiring specialized equipment, computing power, and skilled human resources in laboratory science, bioinformatics, and data analysis. Procurement of specialized reagents and consumables is challenging because of global supply chain shortages and limited local suppliers, and funding is often concentrated in regions where resistance markers have already been detected.^6^^,^^7^ As a result, MMS efforts are frequently project-based, geographically uneven, and weakly integrated into routine surveillance systems.

A regional approach to MMS offers unique solutions to the longstanding challenges of in-country platforms.^8^ Establishing regional sequencing hubs in countries with existing infrastructure can address resource disparities^6^ while optimizing equipment and human resource requirements.^9^ Coupling this approach with standardized sample collection and analytical protocols ensures that MMS are comparable across countries, enabling coordinated intra- and inter-country action. Regional collaborations provide opportunities for pooled procurement and more cost-effective generation of regionally representative MMS data to support timely risk assessment and decision-making at both country and regional levels.

A regional approach is often epidemiologically necessary to effectively understand and contain parasite populations and resistance threats. Human movement across countries and regions connects parasite populations with diverse genetic backgrounds and can facilitate the spread of drug-resistant parasites across borders.^5^ In southern Africa, high population movement, frequent malaria outbreaks, and historical selective sweeps observed in relation to sulfadoxine–pyrimethamine (SP)^10^^,^^11^ underscore the risk of rapid expansion of drug-resistant parasites. This threat, together with the recent de novo emergence and spread of parasites partially resistant to artemisinins across Africa,^12^ further highlights the urgency of generating near-real-time, accurate MMS data.

The processes, successes, challenges, and lessons learned from developing and implementing the Genomics of Malaria in the Elimination Eight (GenE8) Initiative, a regional MMS project in southern Africa, are described in the present study. The authors’ intent is both to document implementation experiences and to highlight transferable lessons for others establishing or managing regional MMS initiatives. A summary of key insights and transferable lessons is provided in Table 1, and a breakdown of sequencing costs, administrative processes, and timelines is listed in Supplemental Table 1.

**Table 1:** Key insights and transferable lessons from the Genomics of Malaria in the Elimination Eight initiative

Domain	What Was Achieved (Outcomes)	Key Constraints Encountered	Implementation Insights and Transferable Lessons
Governance and design	Strong regional coordination and NMP ownership enabled the adoption of the GenE8 MMS model and protocol across the five participating countries.	Limited long-term financing constrained sustainability and follow-up activities.	Regional governance structures are essential for alignment and uptake but must be paired with sustained financing and clear transition plans to support long-term implementation.
Sampling and data systems	Standardized protocols enabled geographically representative sampling and the generation of comparable data across countries.	Weak routine data systems and inconsistent sample quality limited data linkage, while delays in ethics approval and shipment limited the efficiency of analysis.	Effective MMS implementation requires investment in routine data capture systems, early regulatory planning, and continuous training to maintain data and sample quality.
Data sharing and coordination	Harmonized workflows and a regional dashboard enabled real-time tracking of sample shipping and data generation, and provided shared visibility of genomic data to support inter- and intra-country data interpretation.	Delays in data-sharing approvals, regular senior leadership turnover, and reservations related to the sharing of genomic and patient data limited timely dissemination.	Data-sharing systems require not only technical platforms but also enforceable agreements, accountability mechanisms, and stable governance to ensure timely access to and use of data.
Laboratory systems and sequencing	A regional sequencing hub enabled rapid, standardized data generation and efficient use of infrastructure across countries.	Inability to retain trained laboratory staff and frequent procurement delays disrupted workflows, resulting in missed milestones and increased dependence on external partners.	Improved efficiency of regional hubs depends on reliable supply chains and a stable, trained workforce; these operational dependencies must be planned for upfront.
Bioinformatics and analytics	Initial workflows and training enabled basic in-country analysis and interpretation of genomic data by NMPs for programmatic use.	Severe shortages in bioinformatics capacity and reliance on external experts limited advanced analyses and sustainability.	Building local analytic capacity and accessible tools is critical; without this, genomic data cannot be translated into actionable insights.
Capacity strengthening and translation	Training and fellowship programs improved NMP engagement and the ability to interpret genomic data.	Limited time, competing priorities, and training needs exceeded the planned scope, constraining independent data use.	Capacity building must be continuous, embedded within programs and local academic systems, and aligned with operational needs to enable sustained generation and use of MMS data.
Regional collaboration and partnerships	Early engagement and co-design facilitated consensus, protocol harmonization, and participation across countries.	Differences in capacity, priorities, and institutional relationships complicated coordination.	Regional approaches can accelerate adoption but require active management of heterogeneity across countries and institutions.
Programmatic impact and sustainability	Malaria molecular surveillance data informed surveillance planning and stimulated demand for further genomic activities across the region.	Lack of sustained funding and limited in-country capacity threaten continued monitoring of resistance markers and scale-up.	For long-term impact, MMS must be institutionalized within NMPs and supported by domestic financing, not solely donor-funded projects.

GenE8 = the Genomics for Malaria in the Elimination Eight Initiative; MMS = malaria molecular surveillance; NMP = national malaria program.

### The GenE8 initiative.

The Elimination Eight Secretariat (E8S), a subsidiary agency of the Southern African Development Community, oversaw a regional malaria elimination initiative across eight southern African countries (Angola, Botswana, Eswatini, Mozambique, Namibia, South Africa, Zambia, and Zimbabwe) collectively known as the Elimination Eight (E8).^13^ The establishment of the E8 was driven by the recognition that the region’s high level of interconnectedness (N. Ruktanonchan, unpublished data) and clustering of malaria transmission along shared international borders^14^ necessitate a coordinated, cross-border response to effectively control and eliminate malaria. To achieve its ministerial mandate, the E8S (guided and supported by a technical committee, various thematic technical working groups, and partner organizations) implemented and coordinated multiple initiatives, including the GenE8 initiative.^15^

The GenE8 initiative was designed to increase regional coordination and collaboration on MMS as a programmatic decision-making tool while addressing critical evidence and capacity gaps in the generation and utilization of parasite genomic data within southern African NMPs and PHRIs. The initiative was codesigned by the University of California, San Francisco (UCSF), the E8S, the National Institute for Communicable Diseases (NICD) in South Africa, and the Africa CDC, with key technical inputs from Johns Hopkins University (JHU) and experts from E8 member states. The GenE8 initiative encompassed several key activities to strengthen regional malaria genomic surveillance in the E8. Five countries participated in all components (Angola, Eswatini, Namibia, South Africa, and Zambia), whereas Mozambique participated only in coordination and capacity strengthening because of a separately aligned data-generation project.

### Key activities of the GenE8.

- Establishing an MMS Coordination Group to serve as a platform for consensus-building and coordination among E8 member states
- Strengthening malaria genomics-specific technical capacity at the NICD, designated as one of the Africa CDC’s regional sequencing hubs in southern Africa
- Launching the Translational Malaria Genomics (TraMGen) Fellowship to enhance the capacity of NMPs in analyzing and applying genomic data for decision-making
- Conducting training workshops on introductory malaria genomics, including MMS use cases and interpretation, to build the capacity of NMPs and PHRI partners to apply MMS data in programmatic decision-making
- Implementing a regional genomics study to generate representative genotyping data across participating E8 countries to address priority programmatic questions, including those around antimalarial drug and diagnostic resistance, parasite movement within and between countries, and variability around vaccine targets, and establish a biobank
- Supporting country-specific genomic projects through competitive funding to facilitate data generation and analysis at the country level

### Successes and challenges.

#### Initiative design and funding.

Regional surveillance initiatives face unique challenges in both design and funding. By nature, they involve diverse groups of stakeholders, often with differing priorities, making coordination and alignment complex. National malaria programs are frequently excluded from the design stage and capacity-strengthening efforts, yet NMPs make or recommend critical national policy and technical decisions that require MMS data. Additionally, securing sustainable funding for regional initiatives remains a significant challenge because few funding mechanisms are specifically designed to support cross-border, multicountry malaria surveillance efforts beyond discrete projects funded by the Global Fund to Fight AIDS, TB, and Malaria and the Gates Foundation, both of which have supported regional MMS efforts.

To the best of the authors’ knowledge, GenE8 is the only initiative in Africa that integrates both genomic sequencing and capacity-building under a regional framework and explicitly focuses the design, implementation, and capacity-strengthening components on NMPs. The governance structure for GenE8 is shown in Figure 1. The core project management team (PMT) was led by the E8S and included representatives from founding partner institutions and rotating NMP representation. The PMT managed the MMS Coordination Group, TraMGen Fellowship, training workshops, and regional and country project implementation teams that designed and managed data-generating projects.

**Figure 1.**
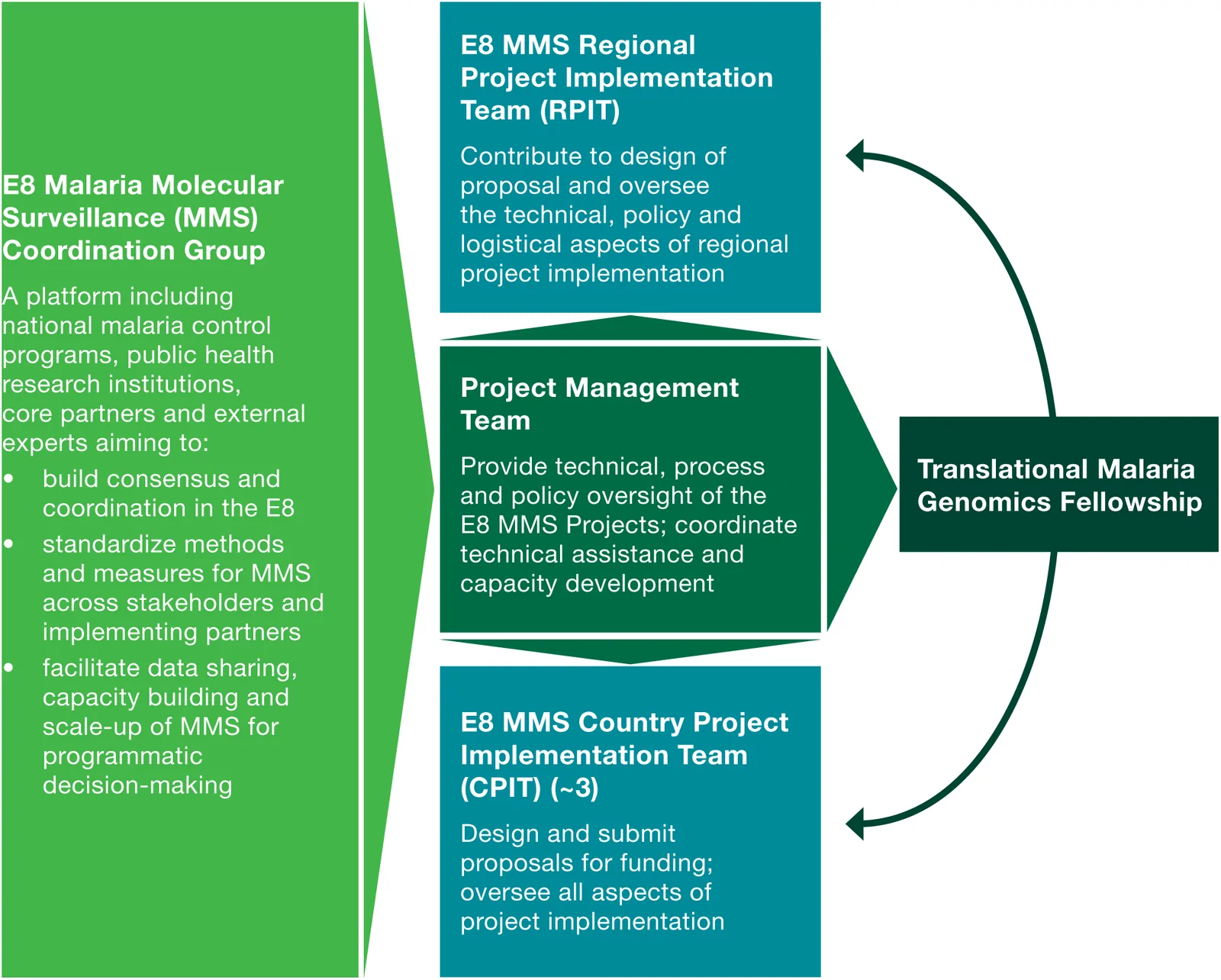
Organization structure of the Genomic for Malaria in the Elimination Eight initiative.

One of the key lessons from GenE8 is that longitudinal data collection and capacity-building efforts, including laboratory training, bioinformatics analysis, and the translation of genomic data into programmatic decision-making, require sustained investment and local supporting structures. Future initiatives should build a funding coalition to secure more sustainable financing for regional genomic surveillance. Although the GenE8 initiative detected high levels of resistance markers in certain areas, there has been no follow-up to this initial investment, risking the rapid spread of resistant parasites with significant increases in malaria-related morbidity and mortality. This is in stark contrast to Southeast Asia, where the critical role of regional approaches in addressing artemisinin partial resistance was promptly recognized, established, and continually funded through the Regional Artemisinin-Resistance Initiative (RAI). With sustained funding and political commitment, the RAI was able to address cross-border human and parasite movement, roll out new treatment regimens and surveillance approaches, facilitate direct coordination between NMPs, and ultimately mitigate the spread of antimalarial drug resistance.^16^^,^^17^

One opportunity to improve the uptake and sustainability of MMS in Africa is to integrate it across disease control programs, enabling the shared use of human and laboratory resources. Without long-term financial support and institutional commitment, the full benefits of MMS for disease control cannot be realized.

#### Sample and data collection.

Passive surveillance remains the most cost-efficient sampling method for MMS. However, not all countries have routine passive surveillance systems in place to consistently collect dried blood spots (DBSs) from confirmed cases, record individual patient data for analysis, including demographic (age, sex, symptomatology, etc.) and travel history data, or systematically enable the linkage of samples with patient data. Operationally, DBSs and patient data must be collected at the point of care before antimalarial treatment is initiated. This is often infeasible in routine surveillance because of overburdened healthcare workers and high staff turnover. Malaria programs in Southeast Asia have effectively addressed their human resource bottlenecks by using community healthcare workers (CHWs).^18^^,^^19^ In Africa, however, the use^20^ and impact^21^^,^^22^ of CHWs are limited. Effective integration of CHWs into malaria surveillance activities would help address human resource challenges, particularly because CHWs are less likely to turnover within the community. However, regular training sessions for CHWs and frequent supervisory support visits to ensure quality data and sample collection would still be required.

Sampling strategies also differ depending on countries’ surveillance goals. In low-transmission settings, for example, understanding detailed genetic epidemiology requires sequencing data from most of the reported cases, whereas broader surveillance of resistant alleles may require sequencing only a subset of infections. The WHO-recommended protocol for the detection of *histidine-rich protein 2* and *3* (*hrp2*/*3*) gene deletions associated with diagnostic resistance further complicates sampling because it requires dual diagnostics for febrile patients, which is impractical in low-transmission rural settings because of large sample size requirements, where test positivity is low and increased costs are associated with the testing of every patient with two different types of rapid diagnostic tests (RDTs).

The GenE8 initiative implemented standardized sample and data collection protocols across all five participating countries, with standardized operating procedures for DBS collection and storage. Sampling strategies were tailored to each country’s needs while aligning with the regional objective of generating geographically representative data on the prevalence and distribution of drug resistance markers. In high-transmission settings, each participating healthcare facility aimed to sample 200 malaria cases (RDT-confirmed infections among febrile patients), targeting at least 50 high-quality samples for sequencing. Low-transmission settings targeted the recruitment of all confirmed malaria cases. A subset of RDT-negative samples from febrile patients found to be malaria-positive by quantitative polymerase chain reaction was assessed for *hrp2*/*3* gene deletions, although the WHO-recommended sample sizes were not met in the present study.

Despite successful standardization, operational challenges emerged. In some instances, linkage of individual patient data to resistance profiles was not possible. This was primarily due to missing or incomplete records associated with paper-based data capture in Angola and inconsistent recording of unique sample barcodes in South Africa. Sample availability and quality varied within and between countries, with insufficient blood volume and fungal contamination from improper DBS drying frequently observed. Additionally, in several instances, the laboratory team observed virtually identical genetic measures, including allele frequencies, across the different samples, suggesting that a single blood sample was used to generate multiple unique patient DBSs. Although no incentives for sample collection were offered, most participating healthcare facilities were provided with a sample target. Achieving this target could have motivated the duplication of samples.

Where possible, existing material transfer agreements (MTAs) were leveraged to streamline and fast-track sample and data transfer. However, slow MTA processing in countries without existing MTAs and limited courier options for biological sample transport in certain countries, where DBS shipments were not exempt from international dangerous goods regulations, led to significant delays in sample shipments.

These challenges highlight the need for an electronic data capture system adapted to resource-limited settings, effective early engagements to ensure that healthcare workers understand MMS principles and processes, and routine training and supervisory visits to ensure proper DBS handling and data collection. The importance of meeting quality standards over sample size must be highlighted during training sessions and emphasized during supervisory visits. To improve MMS efficiency amid resource constraints and declining malaria burden in some countries, NMPs should leverage existing MTAs and shipping mechanisms established for other infectious disease programs (e.g., HIV, COVID-19, tuberculosis). Protocols for *hrp2/3* surveillance require modification to be feasible for low-transmission and outbreak settings.

#### Data standardization and sharing.

Data standardization and timely data sharing are crucial for monitoring threats to malaria control and elimination and coordinating malaria responses across countries. However, differences in genotyping methods, resistance markers, and analytic approaches complicate standardization across projects and countries. The unexpectedly high turnover among senior NMP management staff caused communication delays, which negatively impacted approval of data releases (initially to the other participating countries and then to regional partners and international bodies). Intense lobbying by the E8S, with support from technical experts, was required before permission for data release was obtained. Reluctance among some NMPs to share the generated genomic data once resistance markers were detected delayed the immediate sharing of aggregate data across the E8 region and with international governing bodies, potentially delaying resource mobilization and mitigation responses. The GenE8 Initiative sought to mitigate these challenges by having regular engagements with the Ministries of Health and NMPs from concept and protocol development to final data dissemination, co-creating initiatives objectives and activities with the NMPs, including a research fellow and principal investigator (PI) from each NMP in the project management and implementation committees, using standardized procedures to generate and analyze genomic data, establishing data-sharing agreements during the design phase of the initiative, and creating an interactive drug resistance dashboard. Generated data were released in stages, first to the NMP and partner PHRI (the in-country data owners), and then to regional partners and external parties, in accordance with a consensus-driven data-sharing agreement.

The interactive dashboard enabled participating countries to monitor progress in sample collection, sample shipment, and sequencing at the regional hub in South Africa in near real time. Critically, the dashboard provided centralized visualizations of the prevalence and spatial distribution of the different malaria parasite species and validated molecular markers of artemisinin partial resistance, chloroquine resistance, and SP resistance. To support data interpretation and decision-making, the dashboard contained programmatically relevant guidance on the resistance markers, which was reinforced during regular technical support meetings. Additionally, all generated data were collated and stored in a centralized electronic database at a secure location, accessible only to the core technical team.

Ultimately, for regional surveillance to be effective, NMPs and Ministries of Health must make greater efforts to adhere to the terms of data-sharing agreements, which ideally are applicable across surveillance projects and research initiatives. The creation of a regional oversight committee could avert some of these challenges. The committee would have the right to approve the release of important resistance data within a prespecified timeframe, while holding NMPs accountable for timely review and approval of data release.

#### Genotyping methods.

African disease control programs face several challenges in generating high-quality genotyping data for programmatic use. Molecular assays differ in their sensitivity and technical requirements. Variable access to assays has resulted in sparse datasets across different African settings, and differing genotyping methods limit comparisons within and across countries for certain use cases. Simpler polymerase chain reaction assays detect only known resistance markers, whereas Sanger and capillary sequencing cannot reliably resolve them in polyclonal infections, in which mixed-strain signals produce ambiguous results or mask minority variants. Additionally, these approaches cannot be used to simultaneously assess large panels of resistance markers. Although NGS has become more affordable and accessible, gaps in capacity to generate, analyze, and interpret highly complex sequencing data persist, limiting its wide-scale uptake. Targeted NGS panels offer significant advantages for regional surveillance by addressing multiple use cases concurrently, as demonstrated in the present initiative.

In line with the Africa CDC model of utilizing a regional sequencing hub, the NICD in South Africa was selected as the GenE8 sequencing hub. Technical support and training were provided by UCSF. Targeted investments in infrastructure and workflow processes enabled standardized laboratory protocols and analytic workflows to be scaled up to meet the initiative’s demands. The centralized approach enabled the rapid generation of genomic data over a short time period (6–9 months) using a robust targeted amplicon NGS assay.^23^ To the best of the authors’ knowledge, this marks the first coordinated effort in the E8 region to generate parasite genomic data specifically for programmatic use, as well as the first regional initiative to produce standardized genomic data in Africa. The initiative’s customized sequencing assay covers multiple use cases and has been successfully implemented in laboratories across Africa, Europe, and the Americas. Harmonized laboratory and bioinformatic methods facilitated the efficient sharing of genomic data and rapid comparison and aggregation of data for visualization, enhancing programmatic decision-making and scientific research.

The success of the GenE8 initiative in generating genomic data within a regional sequencing hub highlights both achievements and ongoing challenges. Although the initiative met its data-generation goals, timelines were affected by laboratory staff turnover and delays in the delivery of specialized reagents and consumables. Highly skilled laboratory technicians are in short supply in the region, posing a major challenge for recruiting and retaining a capable workforce. Most NGS reagents are supplied by a single vendor in South Africa, which must import materials from the United States and Europe, causing significant supply bottlenecks. Addressing these challenges requires two solutions: 1) academic and institutional interventions to strengthen laboratory workforce numbers, skills, and retention, and 2) regional and continental systems to streamline and pool procurement for laboratory commodities, ensuring reliable and timely delivery. Service-level agreements with service providers should stipulate that essential reagents and consumables are kept in stock. In addition, based on the experiences of the present initiative, it is essential to have a core set of trained staff at all levels, from data generation through to analysis, who are ideally permanent staff or employed on long-term contracts to reduce disruptions caused by frequent staff turnovers. Trained staff could support multiple disease areas, thereby minimizing initial investments and reducing delays when launching new projects and initiatives.

#### Bioinformatics and data analysis.

Investments in laboratory infrastructure and robust workflow development have improved genomic data generation across the continent. However, raw sequencing data must be processed through bioinformatics pipelines to remove false sequences, enable alignment with known genomes, and accurately characterize genetic variation. Additionally, advanced data analytics are critical for summarizing findings, interpreting results, and inferring population characteristics to guide decision-making. Furthermore, the complexity of the malaria parasite’s biology and life cycle demands unique specialized bioinformatic and analytical tools. There is, however, a critical shortage of skilled bioinformaticians in southern Africa, with many of them based in academic institutions and focused on nonmalarial diseases. Additionally, many epidemiologists and statisticians within NMPs and PHRIs lack expertise in genomics and malaria genetics, hindering their active participation in data analysis. Skills gaps in advanced computational and statistical methods have created significant barriers to translating malaria genomic data for decision-making.

Therefore, in addition to the TraMGen fellowship, two trainees, supported by country-specific projects and technical mentorship, were capacitated to assist with basic downstream analyses, including assessing the prevalence of resistance markers. Additionally, modularized semiautomated bioinformatics workflows were developed to streamline the processing of raw sequencing data, the generation of quality reports, and the formatting of data for downstream analysis. Despite these interventions, certain gaps in computational and analytical capacity persisted, requiring significant bioinformatics and data analysis support from UCSF and JHU.

To reduce reliance on experts from the United States and Europe and enable autonomous data analysis and utilization, future initiatives should be focused on building local capacity and improving access to analytical tools. Strengthening local expertise in bioinformatics and data analysis is essential and can be achieved by equipping existing professionals with domain-specific skills, leveraging regional initiatives to coordinate training efforts, and developing curricula within data science undergraduate and graduate programs in Africa that focus on building these specific skills. Establishing a cadre of highly specialized experts will be critical to supporting the region’s malaria genomics needs. Investments should also be directed towards enhancing tool accessibility by developing user-friendly bioinformatics solutions, leveraging rapid advancements in artificial intelligence, and adopting cloud-based platforms. These advancements would enable laboratorians and end users with limited expertise in bioinformatics or data science to extract meaningful insights from their genomic data.

#### Capacity strengthening and translation of MMS for programmatic decision-making.

Historically, the generation and use of genomic data have been largely confined to academic institutions for research purposes.^24^ However, with the increasing availability and relevance of these data, there is a need to build capacity within NMPs and their partner PHRIs to interpret and apply genomic data effectively. Despite this need, few training initiatives explicitly address translational skills that bridge research findings to decision-making processes within NMPs.

The GenE8 initiative included two capacity-strengthening components directed towards NMPs and PHRI partners in the E8 region. First, annual workshops for NMPs and country PIs provided foundational training in molecular methods, genomic epidemiology, and their practical applications. The second was the TraMGen Fellowship, a year-long program that targeted fellows from the five countries participating in the Regional GenE8 Project, as well as Botswana, who were nominated by their respective NMPs. In addition to building translational skills, the fellowship included hands-on wet laboratory training at the regional sequencing hub in South Africa, in-person and online instruction in epidemiology and introductory malaria genomic data analysis, and individualized mentorship from technical experts. Through the mentorship program, participants were supported in writing their manuscripts and in developing their career paths. Fellowship participants have presented findings at national and international conferences, contributed to manuscript drafting, and led discussions on next steps in MMS in their respective countries.

The GenE8 capacity-strengthening component was designed to complement the Africa CDC MMS laboratory and bioinformatics techniques training program by focusing on developing translational skills. Unfortunately, the Africa CDC MMS training program was refocused to meet critical laboratory gaps during the COVID-19 crisis, ultimately limiting the intended synergy between the two curricula. The GenE8 initiative attempted to respond to this misalignment by expanding the scope of its training programs, including adding wet laboratory training as noted above, but lacked sufficient resources (financial, technical expertise, and the required infrastructure) to robustly address the gaps.

Despite these challenges, the GenE8 program successfully fostered engagement in MMS across the E8 region and empowered NMPs to interpret and act on genomic surveillance findings. However, E8 NMPs face two constraints to the sustained use of MMS as a programmatic tool moving forward. First, NMPs often have high staff turnover, leaving small teams of core workers who must juggle multiple responsibilities and risk losing team members who were trained on MMS and led MMS translation activities within the NMP. Second, genomic expertise within NMPs and Ministries of Health remains scarce, and programs face challenges, including limited time, capacity, and funding to maintain and operationalize genomic surveillance platforms. Cross-cutting laboratory initiatives in public health laboratories working across diseases have been proposed as one solution. Addressing these challenges will be critical for the sustained use of MMS within malaria control programs.

#### Consensus and relationship-building.

The successful implementation of the GenE8 initiative relied on the collaboration of diverse stakeholders, including NMPs, academic and technical experts, funders, and implementing partners, each with differing priorities, needs, and capacities. Achieving consensus around priorities, activities, and protocols was complicated by differing priorities between low and high-transmission countries, varying levels of familiarity with genomic workflows and data, heterogeneity in genomic analytic capacity and infrastructure, and inconsistent working relationships between NMPs and PHRIs.

To navigate these challenges, the GenE8 initiative prioritized early engagement and co-design with the E8S. Leveraging the Secretariat’s leadership, existing regional infrastructure, and cross-border surveillance experience, the initiative brought stakeholders together to build consensus around MMS as a programmatic tool. The E8 MMS Coordination Group workshops included NMPs and PHRIs from all E8 countries, except Zimbabwe (by its own choice), as well as local, regional, and international technical experts, funders, and implementing partners. These workshops were focused on identifying shared priorities for capacity building, data generation, sharing and visualization, and protocol harmonization.

The early engagement strategy and consensus-building workshops successfully facilitated the prompt adoption and implementation of the genomic surveillance protocol across all five participating E8 countries in the data generation component. Priorities were collectively set, protocols were harmonized, and the regional networks were effectively leveraged to accelerate progress. However, achieving consensus on training objectives proved more challenging. Familiarity with genomic workflows and data varied significantly among the NMPs, making it extremely challenging to develop a single genomic training program that met the needs of all NMPs and fellows. Although the GenE8 initiative expanded its intended translational skills training to include aspects of laboratory training, there were insufficient resources to address all identified gaps. This was further compounded by the variability in the working relationships between NMPs and PHRIs. Unfortunately, strengthening these fragile working relationships was not addressed in the present study. However, seconding skilled personnel from PHRIs or other academic partners to NMPs and formalizing collaborations between PHRIs and NMPs could strengthen and formalize sustainable working relationships. Centralized regional or peer-to-peer learning across borders should be prioritized and encouraged to facilitate skill-sharing among countries with varying capacities and resources.

### GenE8 initiative impact.

The MMS translational capacity within participating NMPs increased markedly after capacity-building and knowledge transfer activities delivered through this initiative, which were the primary intended outputs of the capacity-strengthening component. The NMPs exhibited the ability to engage with and interrogate summary genomic data to inform programmatic decision-making. The NMPs from Angola, Namibia, and Zambia have subsequently planned additional studies (therapeutic efficacy studies or additional MMS) in areas where artemisinin partial resistance markers were detected to elucidate the clinical relevance of the detected mutations and to monitor changes in mutation prevalence and distribution. Although Botswana was not involved in data generation, the high prevalence of artemisinin partial resistance mutations in neighboring countries elicited an urgent request for MMS training and support with MMS data generation. Encouragingly, despite opting not to participate in the initiative, Zimbabwe began investigating options to assess the prevalence of artemisinin partial resistance mutations in regions neighboring Zambia after an E8 regional data dissemination meeting.

At the time of writing, it is unclear the extent to which planned follow-up activities will be operationalized because of the drastic cuts in donor funding in 2025.^25^ Acknowledging the value of MMS, NMPs from Angola, Botswana, Namibia, and Zambia, with technical support from the E8S, UCSF, and London School for Tropical Medicine and Hygiene, are drafting an international grant application to support regional MMS, including retaining technical expertise at the regional hub. However, the authors acknowledge that dependence on international donor funds does not build sustainability and that it is imperative that MMS becomes a core activity of NMPs, funded and implemented domestically.

## CONCLUSION

Despite numerous challenges, the GenE8 initiative clearly revealed the feasibility and value of regional malaria genomic surveillance, generating the first dataset on partial artemisinin resistance for several participating countries. By aligning the initiative’s objectives with the malaria genomic surveillance priorities of E8 NMPs, the initiative ensured strong country participation and ownership. As a result, genomic data on the prevalence and distribution of molecular markers of antimalarial drug resistance were generated and shared across borders, providing critical insights to support programmatic decision-making at a national and regional level. Critically, through the capacity and knowledge transfer fostered by the GenE8 initiative, the NMPs were able to engage with and use the generated MMS data to inform programming decisions for surveillance and malaria case management activities.

With the increasing threat of partially artemisinin-resistant parasites becoming entrenched in southern Africa, securing sustainable financing to enable the integration of genomic surveillance into routine malaria monitoring is essential. Investments made through the GenE8 initiative have laid a strong foundation, but long-term success will require strengthening regional sequencing hubs, expanding in-country sequencing capacity, and institutionalizing genomic surveillance within NMPs with the support of PHRIs. Establishing reliable funding mechanisms is key to ensuring that genomic surveillance remains a core component of malaria response efforts, enabling the timely detection and containment of emerging resistance threats.

## Supplemental Materials

10.4269/ajtmh.25-0130 Supplemental Materials
