# Supplementary material for: A Regional Approach to Malaria Molecular Surveillance in Southern Africa: Lessons from the GenE8 Initiative
Source: Am J Trop Med Hyg. 2026 Jul 14;115(5 Suppl):77–84. doi: 10.4269/ajtmh.25-0130 (PMC13736883; doi:10.4269/ajtmh.25-0130)
Supplement: Supplementary file 1 [file tpmd250130.SD1.pdf]

Supplementary Table 1: A breakdown of administrative processes and timelines, and costs associated with data generation and conferencing.

	**Angola**	**Eswatini**	**Namibia**	**South Africa**	**Zambia**
**Date IRB approval from the Ministry of Health was received**	21 December 2022	21 March 2023	22 April 2022	Existing IRB approval utilized	Existing IRB approval utilized
**Date IRB approval from the Public Health Research Institute was received**	N/A	N/A	24 August 2022	Existing IRB approval utilized	Existing IRB approval utilized
**Date budgets and subcontracting completed**	November 2022	Funds managed centrally	January 2023	Funds managed directly	Funds managed centrally
**Total budget for data collection and supervision**	Data currently not available	Data currently not available	Data currently not available	Funded by NMP	Data currently not available
**Number of sites/Healthcare facilities**	16	80	16	127	67
**When the Training of the trainers and data collectors took place**	March 2023	March 2023	March 2023	January 2022	March 2023
**Period for Sample and Data collection**	May-August 2023	March-December 2023	April 2023-December 2023	January 2022-December 2023	April- August 2023
**Number of RDT-positive samples collected**	2030	528	443	4 569	4 997
**Number of samples DNA was extracted from**	1 656	513	274	4 569	4 737
**Number of samples prepared for next-generation sequencing**	1 024	477	264	2 405	3 084
**Number of samples with good-quality sequence data**	1 001	453	264	1 791	2 705

**Dashboard access for country teams to track recruitment, sample processing, and preliminary analyses**	May 2023	May 2023<br>Source (DHIS2)	April 2023	May 2023<br>Source DHIS2	April 2023
**Cost per sample from sample collection to library prep at the NICD**	$60				
**Cost per sample sequenced at the NICD**	$30				
**Cost of Lead Scientist at the NICD (per annum)**	$66 666				
**Cost of a laboratory scientist at the NICD (per annum)**	$32 555				
**Cost of Lead Epidemiologist at the NICD (per annum)**	$66 666				
**Cost of Lead Bioinformatics Specialist at the NICD (per annum)**	$66 666				
**Cost of Database Manager at the NICD (per annum)**	$44 444				
**Conferencing cost of a 5-day training workshop for PIs and fellows (20 delegates) in South Africa with translation**	$11 000				
